# Synthetic hourly electricity load data for the paper and food industries

**DOI:** 10.1016/j.dib.2021.106903

**Published:** 2021-02-20

**Authors:** Javier Valdes, Luis Ramirez Camargo

**Affiliations:** a Institute for Applied Informatics, Technologie Campus Freyung, Technische Hochschule Deggendorf, Freyung, Germany; b Institute for Sustainable Economic Development, University of Natural Resources and Life Sciences, Vienna, Austria; c Electric Vehicle and Energy Research Group, Mobility, Logistics and Automotive Technology Research Centre, Department of Electrical Engineering and Energy Technology, Vrije Universiteit Brussel, Brussels, Belgium; d Flanders Make, 3001, Heverlee, Belgium

**Keywords:** Electricity load profiles, Load shifting, Demand response, Cluster analysis

## Abstract

The data set includes hourly time series for a period of one year of electricity demand of three different types of industries. The industries include a small food processing company, one small paper industry and one medium-large paper producing company. The data have been synthetized from two years of measured data from industries in Chile using a comprehensive clustering analysis. The synthetic data possess the same statistical characteristics as the measured data but are provided normalized to one kWh and anonymized in order to be used without confidentiality issues. The data were originally used in the associated paper [1] to assess the demand side management potential of the industries but these can be used for further energy system modelling exercises including these types of industries.

## Specifications Table

**Table:** 

Subject	Energy
Specific subject area	Electricity demand side management of industries
Type of data	Table
How data were acquired	The raw data was acquired through a cooperation agreement with three companies in Chile. The companies reported the use of smart meters for their total electricity demand from the electrical grid. The derived data (described here) is the result of a thorough analysis that includes preprocessing to eliminate outliers and the application of a series of clustering algorithms to create synthetic electricity demand time series for the individual industries. The entire analysis was conducted with the statistical software R.
Data format	Raw analysed
Parameters for data collection	The conditions for the raw data collection were the existence of cooperation agreement with the companies as well as the availability of measured electricity demand data for at least one year in intraday temporal resolution as well as having and energy management certification in place. Therefore, the data collection was performed directly by the companies. The data were posteriorly shared with the authors.
Description of data collection	The raw data (not provided here) were collected for the period from 1th of January 2015 till December 31st, 2017, in three different industries in Chile. The industries include one small food processing company, one small paper industry, and one medium-large paper producing company.
Data source location	Institution: Technische Hochschule Deggendorf City: Deggendorf Country: Germany Primary data sources: one small food processing company, one small paper industry, and one medium-large paper producing company located in Chile. Names and locations of the companies cannot be provided due to confidentiality agreements.
Data accessibility	Repository name: Mendeley Data Data identification number: https://doi.org/10.17632/ttx9chkdcg.1 Direct URL to data: https://data.mendeley.com/datasets/ttx9chkdcg/draft?a=674b88bd-e334–4e86–8514-ebe2cbc9990d Access to raw data for non-commercial use can be provided on request. To request access to the raw data please contact the Corresponding author(s)
Related research article	Valdes J., Masip Macia Y., Dorner W., Ramirez Camargo L., Unsupervised grouping of industrial electricity demand profiles: synthetic profiles for demand-side management applications, Energy, 215, 2021, 118962, https://doi.org/10.1016/j.energy.2020.118962

## Value of the Data

• The data provided are hourly electricity load profiles for the paper and food industries for one year. Such profiles are scarce and otherwise only available through direct confidentiality agreements with companies.
• Data can be used by engineers and scientist for energy modelling purposes.
• We provide minimal, maximum and mean consumption values for a whole year that can either be directly used after scaling by a desired demand size (each hourly entry has been normalized in values between o and 1) or can be used as a basis for to calculate randomized experiments based on confidence intervals.
• Due to the expansions in the use of smart meters we have seen an increment in open access availability of electricity loads data from residential users. This is however not the case for industrial loads. With the proposed methodology and by making the data available we hope to motivate other researchers to also make their industrial loads data available for the energy modelling community.

## 1 Data Description

Three CSV files are provided: food_i.csv, paper_i_small.csv and paper_i_large.csv containing the data of the small food processing industry, the small paper industry, and the medium-large paper industry, respectively. All the three files contain seven columns of data: weekday, month, hour, cluster, min, max, mean. The four first columns index the data in the following way:

Month: it includes the range of integer values between 1 and 12 accounting for the consecutive calendar months of a year starting in January (1) and ending in December (12).
Weekday: this column has integer values in the range 1 to 7 that are equivalent to the consecutive days of the week starting on Monday (1) and ending on Sunday (7).
Hour: it consist of integer values ranging between 1 and 24, which describe the hours of a day.
Cluster: The column “cluster” represents the cluster to which this data is associated to. The number of clusters is different for each load profile, as well as the number of days included in each cluster. Since the cluster were calculated for days, a cluster number covers 24 consecutive points of data.
The load profile data are provided in the three different columns: min, max and mean:
Min: this column provides the min value of the cluster at that time of the day. Therefore, it represents the minimum demand of electricity recorded in all the days belonging to this representative group of data.
Max: This column provides the maximum electric load of the cluster at that time of the day. It represents the maximum demand of electricity in all the days belonging to this representative group of data at that hour of the day.
Mean: This column provides the average electric load of the cluster at that time of the day. It represents the mean demand for electricity belonging to this representative group of data at that hour of the day.
The min, max and mean values are different for each hour of the day. All values are provided in values from 0 to 1 with the unit kW. To generate new randomized values is necessary to follow the methodology described in the next section.

**Fig. 1:**
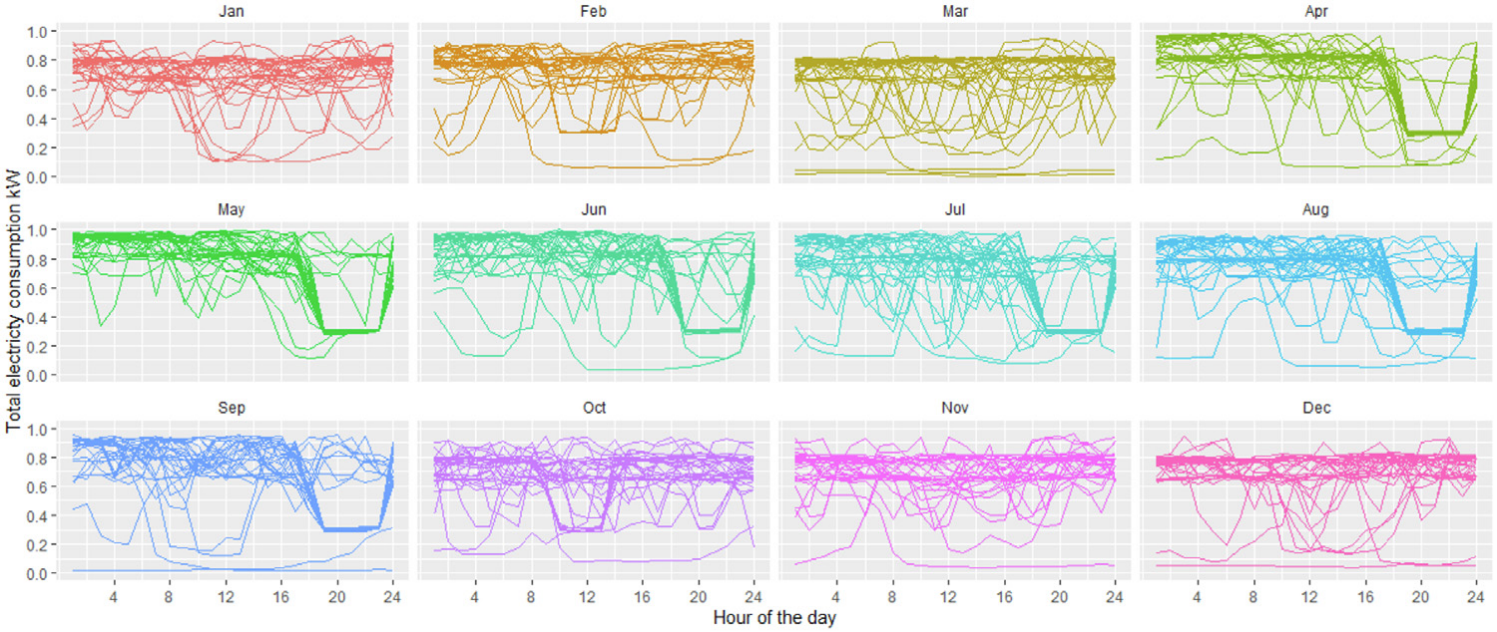
Total electricity demand industry pulp and paper – medium size.

**Fig. 2:**
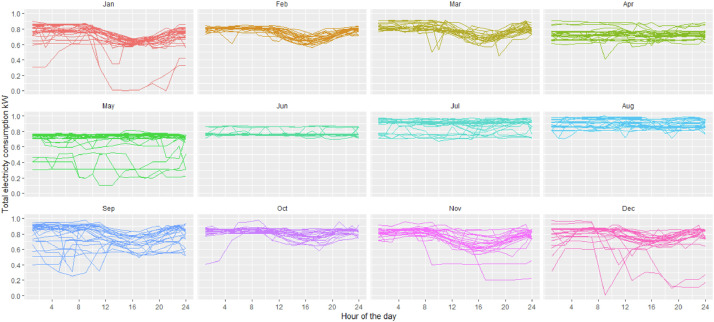
Total electricity demand industry pulp and paper – large size.

**Fig. 3:**
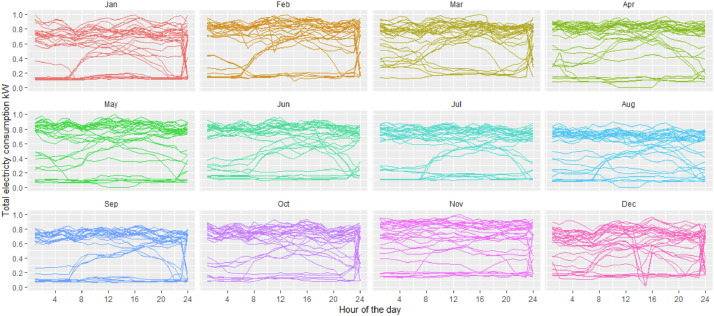
Total electricity demand food industry.

Figs. 1, 2 and 3 show the data grouped by day and month for the small paper industry, the medium-large paper industry and the small food processing industry respectively; each of the lines presents the total energy demand. These plants differ in the size of the demand and the production systems. Both paper and pulp plants have a continuous shift from Monday to Sunday. Both have paper and pulp machines. The large company has sufficient power generation capacity to cover the entire demand for energy and has a network connection for the sale of surplus on the market. The sale of this surplus is not represented in the demand we provide here. The small paper company has a generation capacity sufficient to cover up to 40% of the company's peak demand. The rest of the energy comes from the electric grid, under a time of use (TOU) rate - different rates depending on the time of day. With higher prices in the winter months in the evening hours (it is important to keep in mind that Chile is in the southern hemisphere, implying that the seasons are opposed to locations in e.g. North America or Europe). The food company covers the electricity demand entirely from the electricity network and with a production that is restricted to the working days (Monday to Friday).

## 2 Experimental Design, Materials and Methods

The raw data, from which the data we offer have been generated, were provided directly by the companies and had the quality requirements of an energy management certification program associated with ISO 50,001. The raw data is bonded to a confidentiality agreement and cannot be shared. The data provided here have been generated using a clustering algorithm that allows keeping the statistical characteristics of the raw data. Synthetic hourly time series for one year were generated for the three types of industries and profiles of minimum, mean, and maximum hourly demand. The approach followed here is based on [1] and [2] and complemented with statistical techniques and a new phase: the generation of random load profiles. The original procedure described in [1] contains six phases: i) data gathering and processing; ii) pre-clustering to prepare the data for statistical analysis; iii) time series analysis; iv) clustering analysis, which is carried out in parallel with the previous phase; v) assessment, in which the results of the previous two phases are compared and vi) potential calculation and profiles development.

The methodology proposed by [2] served as a basis and is extended by the introduction of a potential estimation phase in [1], wherein regression techniques are used to verify the consistency of the selected clusters. One of the main problems faced by energy system modelers is the lack of energy consumption data of industries. Companies, usually consider that making these data public can cause problems since it can be used to undermine competitiveness by other companies in the same market. This issue relates to the lack of residential consumption data due to privacy issues. Due to these limitations, several working groups have been working in parallel on the characterization of consumer profiles for different types of consumers. For example, the work of [3] and [4] has recently applied clustering techniques to characterize the demand profiles of different types of households. The techniques they use are similar to those used by [1] and [2] and on which this work is based.

The clustering techniques applied to the original data contain a subgroup of clustering sequences algorithms which is developed in a variety of application fields, including the development of non-redundant databases, function prediction, natural language processing, and even to identify patterns of electricity consumption. As clustering is a type of unsupervised learning, along with clustering techniques, time series analysis has been applied to establish benchmarks for the clustering analysis. Time-series clustering is an active research area with applications in a wide range of disciplines and usually has one or more of the following objectives: data reduction, hypothesis generation, hypothesis testing, or cluster-based prediction [5].

**Fig. 4:**
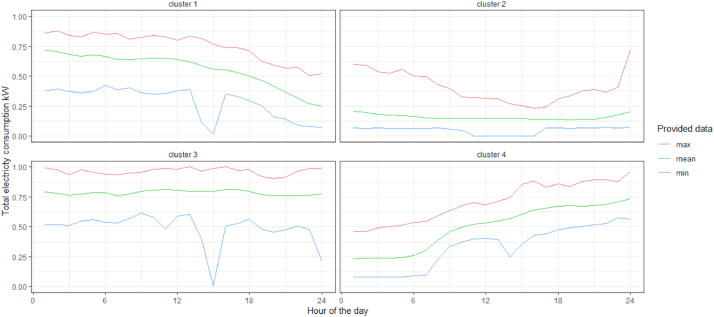
Provided data for the food industry.

The time series can be used directly as there are provided in the files; for this, it is only necessary to decide what type of scenario is going to be simulated (example of options are presented in Fig. 4). The “mean” column provides a scenario with an average annual consumption similar to that of the original series. This series can be employed as a reference or first approximation. The “minimum” series can be used for the calculation of scenarios with a demand considerably lower than the total annual demand. This is useful to simulate e.g. the effect of underutilization of installed capacity. Finally, the “maximum” time series allows the calculation of scenarios with a demand considerably higher than the total annual demand. This kind of scenario allows the simulation of scenarios where e.g. peak demand or operational limits are relevant. The data provided here have to be scaled to the size of the company of interest. Since all values are provided in the range between 0 and 1, it is enough to multiply each entry in the time series by the maximum demand value in order to obtain the load profile of the studied company. This maximum demand value can be obtained for example from the contracted maximum load with the electricity provider of the company or by knowing the installed capacity of the electricity generation technology in case the company is self-sufficient.

**Fig. 5:**
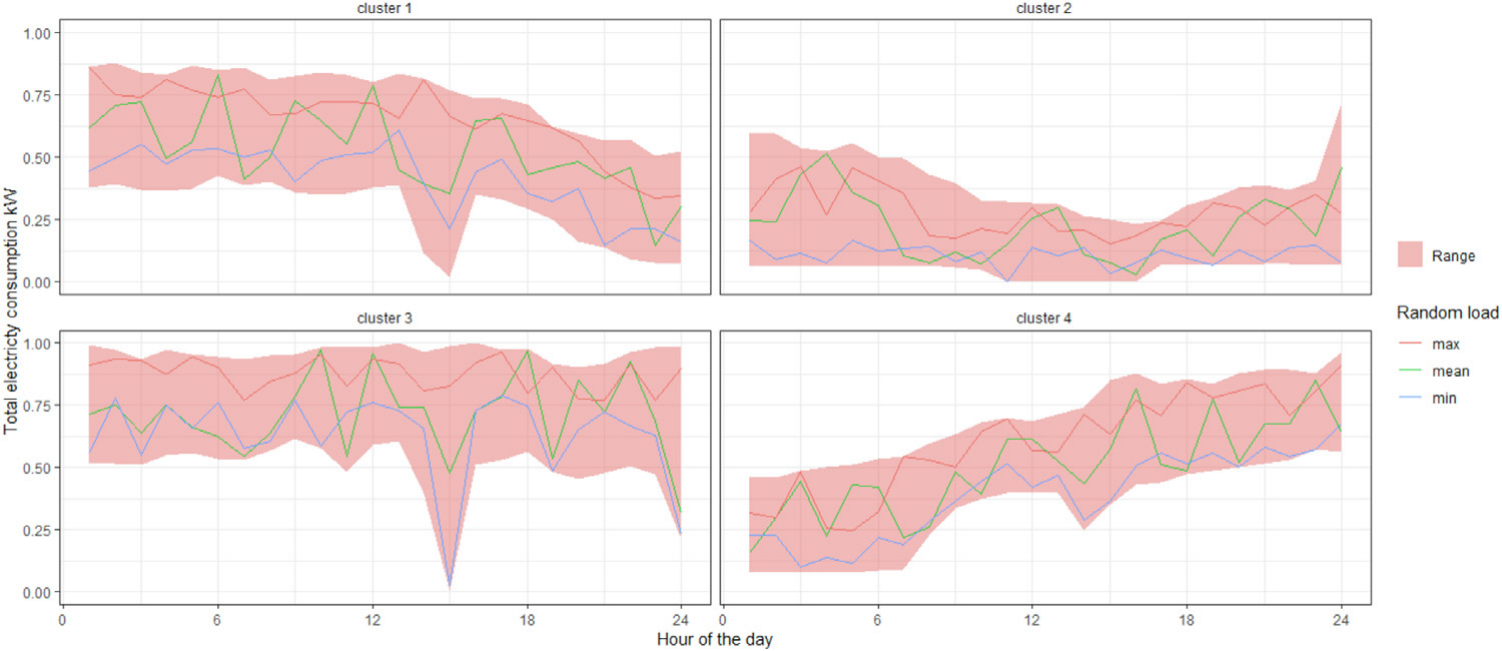
Example of random loads based on provided data for the food industry.

Together with the use of the demand data we directly provide, it is possible to generate new demands from the range that exists between the “maximum” and “minimum”. By Generating random values it is possible to generate a synthetic demand with demand entries for each hour between the minimum and maximum ranges, as in Fig. 5. These new load profiles allow to add an element of dynamism to the series we offer here. Besides, it allows us to combine elements such as peaks and valleys randomly, but always within a representative range of consumption. Finally, using such an approach iteratively, it is possible to generate risk assessments and to create more complex constructions as confidence intervals.

## Declaration of Competing Interest

The authors declare that they have no known competing financial interests or personal relationships which have, or could be perceived to have, influenced the work reported in this article.

## Data Availability

Hourly electricity load profiles of paper producing and food processing industries (Original data) (Mendeley Data).

## Appendix Supplementary materials

Image, application 1
